# Nestin positively regulates the Wnt/β-catenin pathway and the proliferation, survival and invasiveness of breast cancer stem cells

**DOI:** 10.1186/s13058-014-0408-8

**Published:** 2014-07-24

**Authors:** Zuowei Zhao, Ping Lu, Hao Zhang, Huanming Xu, Ningning Gao, Man Li, Caigang Liu

**Affiliations:** 1 Department of Breast Surgery, the Second Affiliated Hospital of Dalian Medical University, 465 Zhongshan Road, Dalian, 116027 Liaoning Province China; 2 Department of Transfusion, the Fourth Affiliated Hospital of China Medical University, 4 Chongshan East Road, Shenyang, 110032 Liaoning Province China; 3 Department of Ultrasonic Diagnosis, the First Affiliated Hospital of China Medical University, 155 Nanjing South Street, Shenyang, 110001 Liaoning Province China

## Abstract

**Introduction:**

We investigated Nestin expression in triple-negative breast cancer and examined how the modulation of Nestin expression affects cell cycle progression, survival, invasion and regulatory signaling in breast cancer stem cells (CSC) *in vitro*.

**Methods:**

Nestin expression in 150 triple-negative breast cancer specimens were examined by immunohistochemistry. The role of Nestin expression in tumorigenesis was examined by assaying naturally occurring Nestin^high^/Nestin^low^ CSC from 12 breast cancer tissues, as well as CSC from 26 clinical specimens, where Nestin overexpression and silencing was achieved by genetic manipulation, for their ability to form mammospheres and induce solid tumors. Cell cycle progression, spontaneous apoptosis and invasiveness of Nestin-silenced breast CSC were investigated by flow cytometry and transwell assays. The relative levels of expression of epithelial-mesenchymal transition (EMT) and Wnt/β-catenin pathway-related molecules were determined by western blotting.

**Results:**

Nestin expression was significantly associated with poor survival in patients with triple-negative breast cancer (*P* = 0.01). Nestin^high^ breast CSC rapidly formed typical mammospheres *in vitro*. Nestin^high^, but not Nestin^low^ CSC, efficiently formed solid tumors *in vivo*. Nestin silencing induced cell cycle arrest at G2/M (52.03% versus 19.99% in controls) and promoted apoptosis (36.45% versus 8.29% in controls). Nestin silencing also inhibited breast CSC invasiveness, and was associated with significantly upregulated E-cadherin, while N-cadherin, vimentin, a-smooth muscle actin (a-SMA), matrix metalloproteinase-2 (MMP-2), MMP-9 and vascular endothelial growth factor (VEGF) expression was downregulated (*P* <0.05 for all). Nestin silencing also upregulated Axin, glycogen synthase kinase-3 beta (GSK-3β), adenomatous polyposis coli (APC), and peroxisome proliferator-activated receptor alpha (PPARa), and downregulated β-catenin, c-Myc, cyclin D and MMP-7 expression in CSC. Inhibition of the Wnt/β-catenin pathway mitigated mammosphere formation in Nestin^high^ CSC, while inhibition of GSK-3β promoted the mammosphere formation in Nestin^low^ CSC (*P* <0.05 for all).

**Conclusions:**

Our data indicates that Nestin positively regulates the proliferation, survival and invasiveness of breast CSC by enhancing Wnt/β-catenin activation.

**Electronic supplementary material:**

The online version of this article (doi:10.1186/s13058-014-0408-8) contains supplementary material, which is available to authorized users.

## Introduction

Breast cancer is the most common malignancy in women, and its incidence is increasing worldwide. The disease remains a huge threat to women’s health. Patients with ‘triple-negative’ breast cancer, referring to absence in the expression of estrogen receptor (ER), progesterone receptor (PR) and human epidermal growth factor receptor 2 (HER2), are insensitive to hormonal therapy or HER2-targeted agents [1]. These patients are also at increased risk for relapse and metastasis of breast cancer, which is often incurable and a leading cause of female mortality [2]–[4]. Unfortunately, there is currently no effective therapy to control the recurrence and metastasis of triple-negative breast cancer. Therefore, understanding the molecular mechanisms underlying the recurrence and metastasis of triple-negative breast cancer may reveal new therapeutic targets, and can significantly improve the management of patients with triple-negative breast cancer.

Nestin, an intermediate filament protein, was initially identified as a neural stem cell marker [5]–[7]. Nestin expression has been detected in malignant tumor tissues, and has been implicated in the development and metastasis of malignant tumors, such as brain malignancies [8],[9], melanoma [10], colorectal [11], prostate [12], and pancreatic cancers [13]. Nestin overexpression has been reported in metastatic breast cancers, (especially in triple-negative breast cancers) [7],[14]–[17], and has been associated with poor prognosis in a Caucasian breast cancer cohort [16]. However, this association has not been verified in Chinese patients with triple-negative breast cancer.

Cancer stem cells (CSC) play an important role in the development and metastasis of breast cancer [18]–[20]. Breast CSC exhibit potent tumorigenicity, and implantation with a few ESA^+^CD44^+^CD24^−^ lineage^−^ breast CSC has been demonstrated to induce solid tumor formation in severe combined immunodeficiency (SCID) mice [21]. Our previous study shows that Nestin and other stemness factors are expressed in breast cancer tissues, and that their expression is associated with poor survival of patients with breast cancer [22]. These findings suggest that Nestin may regulate the development and metastasis of breast cancers. However, little is known about the molecular mechanisms by which Nestin regulates cell proliferation, survival, and invasiveness of breast CSC. Previous studies have shown that the Wnt/β-catenin pathway is crucial for the development and progression of breast cancer [23],[24]. High levels of Wnt receptor and co-receptor expression, as well as aberrant activation of β-catenin, have been detected in breast cancer tissues. Downregulation of the Wnt/β-catenin pathway can inhibit the epithelial-mesenchymal transition (EMT) and reduce spontaneous invasion of breast cancer cells [25],[26]. Therapeutic targeting of the porcupine (PORCN) protein suppresses Wnt/β-catenin activation, and significantly reduces the spontaneous development of mammary tumors in transgenic mice [27]. In addition, aberrant activation of the Wnt/β-catenin pathway has been associated with the development of resistance to radiotherapy and chemotherapy in breast cancer [28],[29]. The Wnt/β-catenin pathway is also important for self-renewal and migration of breast CSC [30],[31]. β-catenin is expressed constitutively in many types of tumor cells. Increased Wnt/β-catenin activation enhances the tumorigenicity of breast CSC [32]. In the absence of Wnt binding to its receptor, β-catenin forms a degradation complex that includes the Axin, adenomatous polyposis coli (APC), and glycogen synthase kinase-3 beta (GSK-3β) proteins. GSK-3β/ casein kinase 1 (CK1) phosphorylates β-catenin, targeting it for β-TrCP1-mediated degradation, which is positively regulated by peroxisome proliferator-activated receptor alpha (PPARa) and PPARb. Binding of Wnt to its receptor and co-receptors causes the activation of Dishevelled (Dsh) proteins by phosphorylation. Activated Dsh then recruits GSK-3β, releasing β-catenin and promoting the nuclear translocation of β-catenin. Subsequently, β-catenin binds to the Tcf and Lef transcription factors in the nucleus, leading to the transcription of downstream genes, including *c-Myc, cyclin D,* and *MMP-7*. Although Wnt/β-catenin activation can promote the expression of various stemness factors, including Nestin, little is known about whether the modulation of Nestin expression can affect Wnt/β-catenin activation in breast CSC.

In the present study, we first characterized Nestin expression in 150 tumor specimens from patients with triple-negative breast cancer, and analyzed the potential association between levels of Nestin expression and the survival of patients. We isolated CD44^+^CD24^−^ CSC from 26 triple-negative breast cancer tissues to generate Nestin-overexpressing (Nestin^+^), Nestin-silencing (Nestin-si), and control (Nestin-c) CSC. We also isolated CD44^+^CD24^−^, cell surface Nestin^high^, or Nestin^low^ CSC from 12 triple-negative breast cancer tissues. Subsequently, we characterized the ability of these CSC to form mammospheres *in vitro*, and of Nestin^high^ or Nestin^low^ to induce tumors in SCID mice. We examined the effects of Nestin silencing on the cell cycle, survival, and apoptosis of breast CSC, and explored potential mechanisms underlying the action of Nestin in the tumorigenicity of breast CSC. Our data suggest that Nestin may promote the proliferation, survival, and migration of breast CSC by enhancing Wnt/β-catenin activation.

## Methods

### Breast tissue specimens

A total of 150 breast cancer samples were obtained from patients with ER-/PR-/HER2- (triple-negative) breast cancer at the Department of Breast Surgery of the First Affiliated Hospital of China Medical University between January 2001 and December 2006. In addition, 12 patients with triple-negative breast cancer were recruited and ESA^+^CD44^+^CD24^−^ lineage^−^ CSC in the resected breast cancer tissues were isolated. The CSC were stained with anti-Nestin and sorted for cell surface positive Nestin^high^ or negative Nestin^low^ CSC. Patients with triple-negative breast cancer were diagnosed by histological examination of tissue samples, and they underwent radical surgery in the Department of Breast Surgery. The inclusion criteria were: (a) curative surgical resection; (b) pathological examination of the resected tumor; (c) pathological examination of >15 lymph nodes after surgery; and (d) availability of a complete medical record. The demographic and clinical data for individual patients were obtained from medical records. Individuals with breast cancer were excluded if they did not fulfill the criteria for inclusion. Written informed consent was obtained from individual patients and the experimental protocol was approved by the Ethics Committee of China Medical University.

### Immunohistochemical staining

Individual breast cancer tissue samples were fixed in 10% neutralized formalin (pH 7.0) and paraffin-embedded. The tissue sections (4 mm) were dewaxed, rehydrated, and treated with 3% H2O2 in methanol, followed by incubation overnight with primary anti-Nestin antibody (Santa Cruz Biotechnology, Santa Cruz, CA, USA). Subsequently, the tissue sections were incubated with Multi-Link biotinylated swine anti-goat/mouse/rabbit immunoglobulin (Ig)G (Dako, Carpinteria, CA, USA). After washing the cells, the bound antibodies were detected using horseradish peroxidase (HRP)-conjugated avidin-biotin complex (1:1000 dilution, Vector Laboratories, Burlingame, CA, USA), and visualized using 3,3-diaminobenzidine (DAB), followed by counterstaining with Gill’s hematoxylin.

The intensity of anti-Nestin staining was scored semi-quantitatively. Cells with yellow to brown cytoplasmic staining were considered to be Nestin^+^ cells. Nestin expression levels in individual tumor tissues were assigned a score based on the following criteria: 0 if <1% neoplastic cells are Nestin^+^; 1+ if neoplastic cells were 1 to 10% Nestin^+^; and 2+ if ≥10% Nestin^+^ neoplastic cells. Individual sections with 1+ or 2+ anti-Nestin staining were considered positive.

### Preparation and characterization of breast CSCs

Thirty-eight freshly resected breast tumor specimens were obtained for the preparation of CSC, as described previously [33]. Briefly, fresh breast tumor specimens were cut into small pieces and were digested with 1 mg/mL of collagenase type III (5 mL/g tissue, Worthington Biochemical, Lakewood, NJ, USA) in 5% fetal bovine serum (FBS) containing RPMI medium at 37°C for 2 h, and centrifuged. The tumor cells (106/tube) were sequentially stained with FITC-anti-CD2, APC-anti-CD3, PE-anti-CD10, FITC-anti-CD16, APC-anti-CD18, PE-anti-CD31, and FITC-anti-CD326 lineage markers, as well as 7-AAD (BD Biosciences Pharmingen, San Diego, CA, USA). Lineage^+^ and dead cells were first eliminated by flow cytometric sorting. Subsequently, the unstained lineage-negative cells were stained with PE-anti-CD24 and FITC-anti-CD44, and CD44^+^CD24^−^ breast CSC were purified by flow sorting. Finally, the sorted CD44^+^CD24^−^ breast CSC were stained with rabbit anti-Nestin (#N5413; Sigma-Aldrich, St. Louis, MO, USA) and then with APC-anti-rabbit IgG to allow sorting of the cell surface positive (Nestin^high^) and negative (Nestin^low^) breast CSC.

### Transfection

The purified CD44^+^CD24^−^ breast CSC from 26 specimens were transfected with human Nestin-specific (sc-36032, a mixture of sc-36032A, sense 5′-CGAGGUCUUUAGAAGAAGAtt-3′ and antisense 5′-UCUUCUUCUAAAGACCUCGtt-3′; sc-36032B, sense 5′-GCCUUUAGAUCUCUAGAAAtt-3′ and antisense 5′- UUUCUAGAGAUCUAAAGGCtt-3; sc-36032C, sense 5′- GGCAAUGAAUCCUCUAGAAtt-3′ and antisense 5′-UUCUAGAGGAUUCAUUGCCtt-3′) or control small interfering RNA (siRNA) (sc-37007, Santa Cruz Biotechnology) using Lipofectamine 2000 Reagent (Invitrogen, Carlsbad, CA, USA), according to the manufacturers’ protocols. Nestin-silenced (Nestin-si) and control siRNA-transfected (Nestin-c) CSC were harvested at 24 or 48 h posttransfection, and the efficacy of Nestin silencing was determined by western blotting. To generate Nestin overexpressing CSC, CD44^+^CD24^−^ cells were transfected with pIRES-Nestin-EGFP (constructed in our laboratory) or control pIRES-EGFP (Clontech Laboratory, Mountain View, CA, USA) for 48 h. EGFP^+^ Nestin^+^ cells were then isolated by flow sorting. A preparation of EGFP^+^Nestin^+^ CSC with a purity of >95% was used for the subsequent experiments.

### Mammosphere formation and *in vivo*xenograft assays

Nestin^+^, Nestin-si, Nestin-c, Nestin^high^, Nestin^low^, and unmanipulated control breast CSC (5,000 cells/mL) were cultured in triplicate in Complete MammoCult™ Medium in six-well ultralow attachment plates (Corning Life Sciences, Tewksbury, MA, USA) for five days in the presence of human leukemia inhibitory factor (LIF, 50 ng/mL), an enhancer of mammosphere formation *in vitro*. The number of mammospheres (>50 mm in a dimension) in individual wells were counted in a blinded manner. In addition, Nestin^high^ CSC in the presence of LGK974 (1 nM), Nestin^low^ CSC in the presence of SB216763 (5 μM, Selleckchem, Houston, TX, USA), and control CSC in the presence of a vehicle were tested for the formation of mammospheres *in vitro*.

Eight-week-old female C57BL/6 SCID mice were obtained from The Jackson Laboratory (Beijing, China) and housed in a specific pathogen-free facility on our campus. C57BL/6 SCID mice were implanted with different numbers (10^3^ to 10^5^/mouse) of the purified ESA^+^CD44^+^CD24^−^lineage^−^ Nestin^high^, Nestin^low^, and control CSC in 50 ml phosphate-buffered saline (PBS) in their mammary fat pads. The development of solid tumors was monitored for up to 28 days post xenotransplantation. The levels of Nestin expression in the dissected tumors were determined by western blotting. The experimental protocol was approved by the Animal Care and Research Committee of China Medical University.

### Flow cytometric analysis of cell cycling and apoptosis in CSC

Following transfection for 96 h, the Nestin-si and Nestin-c breast CSC were fixed by treatment with 70% cold ethanol, followed by staining with propidium iodide (PI, 30 μg/mL). The percentages of CSC in each phase of the cell cycle were determined by flow cytometry on a FACS Calibur device (BD Biosciences, San Jose, CA, USA). The Nestin-si and Nestin-c breast CSC were stained with FITC-Annexin V (2.5 μg/mL) and PI (5 μg/mL), and the percentages of spontaneously apoptotic Nestin-si and Nestin-c breast CSC were determined by flow cytometry.

### Transwell invasion assay

Nestin-si and Nestin-c breast CSC (1 × 10^5^ cells/well) were cultured in triplicate at 37°C for 1.5 h in the upper chambers of transwell plates (Corning) that had been loaded with 60 to 80 μL of diluted Matrigel (BD Biosciences). The cells that had migrated into the lower chamber were fixed with 95% ethanol and stained with crystal violet in gluteraldehyde, followed by visualization. The number of migrated cells in 10 random fields (magnification 400×) of each chamber were counted in a blinded manner.

### Quantitative reverse transcription PCR

The levels of Nestin, Axin, GSK-3β, APC, and β-catenin mRNA transcripts relative to β-actin were determined by quantitative reverse transcription PCR (qRT-PCR). Briefly, total RNA was isolated from Nestin-si and Nestin-c CSC using Trizol reagent, and reverse-transcribed into cDNA using the RevertAid™ First Strand cDNA synthesis kit (Fermentas, Pittsburgh, PA, USA), according to the manufacturer’s instructions. The real-time PCR was performed using the SYBR Green PCR Master Mix and specific primers (Table 1) on an Applied Biosystems 7500 Fast Real-Time PCR System (Applied Biosystems, Carlsbad, CA, USA). The amplification was performed at 95°C for 5 min and then 40 cycles of 94°C for 15 s, 55°C for 20 s, 72°C for 20 s, followed by 72°C for 7 min. The levels of mRNA transcripts were analyzed by the 2-ΔΔCt method. The relative levels of each gene transcript relative to β-actin in Nestin-c CSC were designated as 1.

**Table 1:** **The sequences of primers**

Genes	Sequences	Sizes (bp)
Axin	F: 5′-CTCCAgTAgACggTACAgCgAAg-3′	90
	R: 5′-gCATAgCCggCATTgACATA-3′	
GSK-3β	F: 5′-CTACCAAATgggCGAGCATGAG-3′	112
	R: 5′-CTGCTTGAATCCGAGCATGAG-3′	
Nestin	F: 5′-CTCCAAGAATGGAGGCTGTAGGAA-3′	75
	R: 5′-CCTATGAGATGGAGCAGGCAAGA-3′	
APC	F: 5′-CCTCTGAAACAGTGCTGAACTTG-3′	158
	R: 5′-CACCTGGTACTTGGCCACTA-3′	
β-catenin	F: 5′-GTACGTCCATGGGTGGGACA-3′	80
	R: 5′-GGCTCCGGTACAACCTTCAACTA-3′	
β-Actin	F: 5′-TGGCACCCAGCACAATGAA-3′	186
	R: 5-CTAAGTCATAGTCCGCCTAGAAAGCA-3′	

### Western blot analysis

Nestin-si and Nestin-c breast CSC were collected 48 h posttransfection and lysed in lysis buffer, followed by centrifugation. After quantification of protein concentrations using a BCA assay (Santa Cruz Biotechnology), the individual cell lysates (30 mg/lane) were separated by sodium dodecyl sulfate polyacrylamide gel electrophoresis (SDS-PAGE) and transferred onto polyvinylidene fluoride (PVDF) membranes. The membranes were blocked with 5% fat-free milk powder in TBST and incubated with rabbit anti-Nestin, mouse anti-GSK-3β, anti-phosphorylated GSK-3β, anti-Axin, anti-APC (1:500; Cell Signaling Technology, Beverly, MA, USA), mouse anti-matrix metalloproteinase-2 (MMP-2), anti-MMP9 (1:400), anti-vimentin, anti-vascular endothelial growth factor (VEGF) (1:800), mouse anti-a-smooth muscle actin (a-SMA) (1:400), goat anti-E-cadherin (1:500; Santa Cruz Biotechnology), rabbit anti-N-cadherin (1:500), and rabbit anti-β-actin (1:5000; Abcam, Cambridge, UK) overnight at 4°C, respectively. After being washed, the bound antibodies were detected by incubation with HRP-conjugated anti-rabbit, anti-mouse, or anti-goat IgG at room temperature for 1 h, and visualized using enhanced chemiluminescence (Santa Cruz Biotechnology). Purified mouse, rabbit, or goat IgG were used as the negative controls. The levels of target proteins relative to β-actin were determined using the ImmuNe software. Furthermore, nuclear and cytoplasmic proteins were extracted from Nestin-si and Nestin-c CSC and subjected to western blot analysis. The relative levels of Nestin expression in the purified Nestin^high^, Nestin^low^, and unsorted control CSC as well as the dissected tumors from SCID mice that had been implanted with Nestin^high^, Nestin^low^, or control CSC were also analyzed by western blotting.

### Statistical analysis

Data are presented as mean ± standard deviation (SD). Difference between groups was analyzed using Student’s *t* test or the chi-square test. The cumulative survival of patients with Nestin^+^ or Nestin^−^ triple-negative breast cancer was estimated using the Kaplan-Meier method, and analyzed using the log-rank test. All the statistical analyses were performed using the SPSS statistics 16.0 software package (SPSS Inc, Chicago, IL, USA). A *P* value of <0.05 was considered statistically significant.

## Results

### Nestin expression is associated with poor survival in patients with triple-negative breast cancer

**Figure 1:**
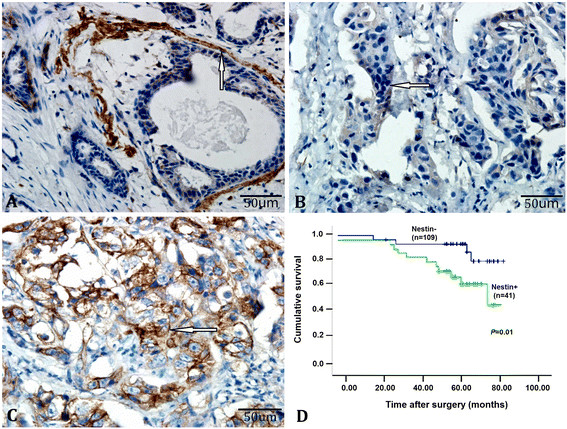
**Nestin expression is associated with poor survival in patients with triple-negative breast cancer.** Nestin expression in 150 surgically resected breast tissue samples from patients with triple-negative breast cancer was examined by immunohistochemistry. Survival was estimated using the Kaplan-Meier method, and the association between survival and Nestin expression was analyzed using the log-rank test. Data are representative images (400× magnification) and the cumulative survival of 150 patients. **(A)** Positive anti-Nestin staining in myoepithelial cells in the surrounding nontumor areas; **(B, C)** negative and positive anti-Nestin staining in breast tumors (white arrow indicates positive cytoplasmic staining). **(D)** Cumulative survival of patients with triple-negative breast cancer.

Nestin is an intermediate filament protein expressed by neural precursors, muscle, and stem cells [34], and has been shown to regulate cell proliferation. Our previous study showed that Nestin is expressed in human breast cancer tissue, and that its expression was associated with lymph node metastasis in a cohort of breast cancer patients [16]. To further understand the role of Nestin in the development and progression of triple-negative breast cancer, we characterized the levels of Nestin expression in 150 specimens from patients with triple-negative breast cancer by immunohistochemistry (Figure 1A-C). Nestin expression was detected in myoepithelial cells in all of the matched adjacent nontumor areas (Figure 1A) and in the cytoplasm of tumor cells in 41 specimens (27.33%, Figure 1B and C), consistent with our previous observations [16]. Survival analysis indicated that the survival of patients with Nestin^+^ triple-negative breast cancer was significantly reduced when compared with Nestin^−^ triple-negative breast cancer (*P* = 0.01, Figure 1D). These results further support a notion that Nestin regulates breast cancer progression, and suggest that Nestin expression may serve as a prognostic marker in patients with triple-negative breast cancers.

### Nestin^high^breast CSC exhibit an enhanced tumorigenicity

**Figure 2:**
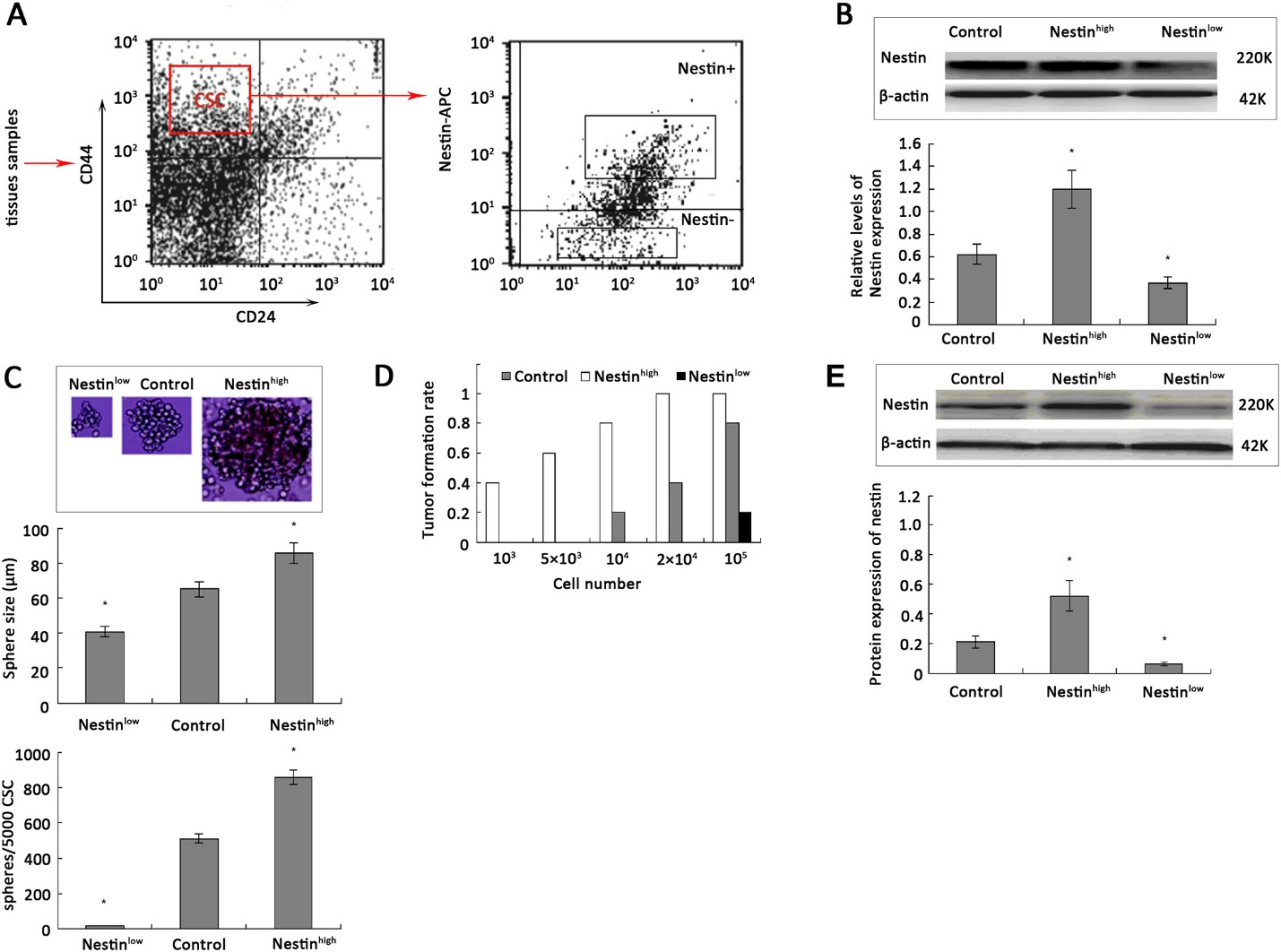
**Nestin**^**high**^**breast CSC have potent tumorigenicity.** The isolated breast CD44^+^CD24^−^ CSC from 12 freshly resected breast cancer tissues were stained with anti-Nestin, and the Nestin^+^ and Nestin^−^ breast CSC were purified as Nestin^high^ or Nestin^low^ CSC by flow cytometry, respectively. The levels of Nestin expression in Nestin^high^, Nestin^low^, and unstained control CSC were determined by western blotting, and these CSC were tested for their capacity to form mammospheres *in vitro* and to induce solid tumors *in vivo*. Finally, the levels of Nestin expression in the solid tumors induced by Nestin^high^, Nestin^low^, or control CSC were determined by western blotting. Data are representative charts and images, and expressed as the mean ± SD of each group of samples (n = 12 per group). **(A)** Flow cytometric analysis of CSC. **(B)** Western blot analysis of the levels of Nestin expression. **(C)** Mammosphere formation. **(D)** The rates of tumor formation in SCID mice (n = 10 animals per group) from three separate experiments. **(E)** Western blot analysis of Nestin expression in the formed tumors (n = 2 to 10 per group). CSC, cancer stem cell; SCID, severe combined immunodeficiency; SD, standard deviation.

Breast CSC express Nestin and are crucial for the development and progression of breast cancer [35]. To understand how Nestin regulates the proliferation and migration of breast CSC, we analyzed the expression of Nestin in ESA^+^CD44^+^CD24^−^lineage^−^ CSC. We found that the proportion of CD44^+^CD24^−^ Nestin^high^ CSC (57.45 ± 18.27%) were significantly higher than that of CD44^+^CD24^−^ Nestin^low^ CSC (42.55 ± 16.4) in the 12 specimens isolated from patients with triple-negative breast cancer (*P* <0.05, Figure 2A). Western blot analysis indicated that the relative levels of Nestin expression in Nestin^high^ CSC were significantly higher than that of control CSC, and significantly higher than that in Nestin^low^ CSC (Figure 2B). Furthermore, Nestin^high^ CSC effectively formed mammospheres, with the mean size and numbers of mammospheres significantly greater than that of the control CSC (Figure 2C). In contrast, the Nestin^low^ CSC failed to form typical mammospheres, with the mean size of mammospheres significantly smaller than that of control CSC. Next, we tested the effect of Nestin expression on the tumorigenicity of breast CSC *in vivo*. We found that implantation of 10^3^ Nestin^high^ CSC resulted in tumor formation in 4 out of 10 C57BL/6 SCID mice, and the rates of tumor formation by Nestin^high^ CSC increased with increasing numbers of CSC, while implantation with 104 control CSC or 105 Nestin^low^ CSC was required to induce tumors (2 out of 10 mice, Figure 2D). More importantly, the relative levels of Nestin expression in the formed solid tumors from the different types of CSC was similar to that of the corresponding type of injected CSC (Figure 2E). These data indicate that Nestin^high^ CSC had at least 100-fold greater tumorigenicity than Nestin^low^ CSC in our experimental system. Taken together, the rapid formation of mammospheres *in vitro* and the efficient induction of solid tumors *in vivo* clearly demonstrated that higher levels of Nestin expression enhanced the tumorigenicity of CSC, which may contribute to the progression and metastasis of triple-negative breast cancer.

### Nestin silencing induces cell cycle arrest and apoptosis in breast CSC

**Figure 3:**
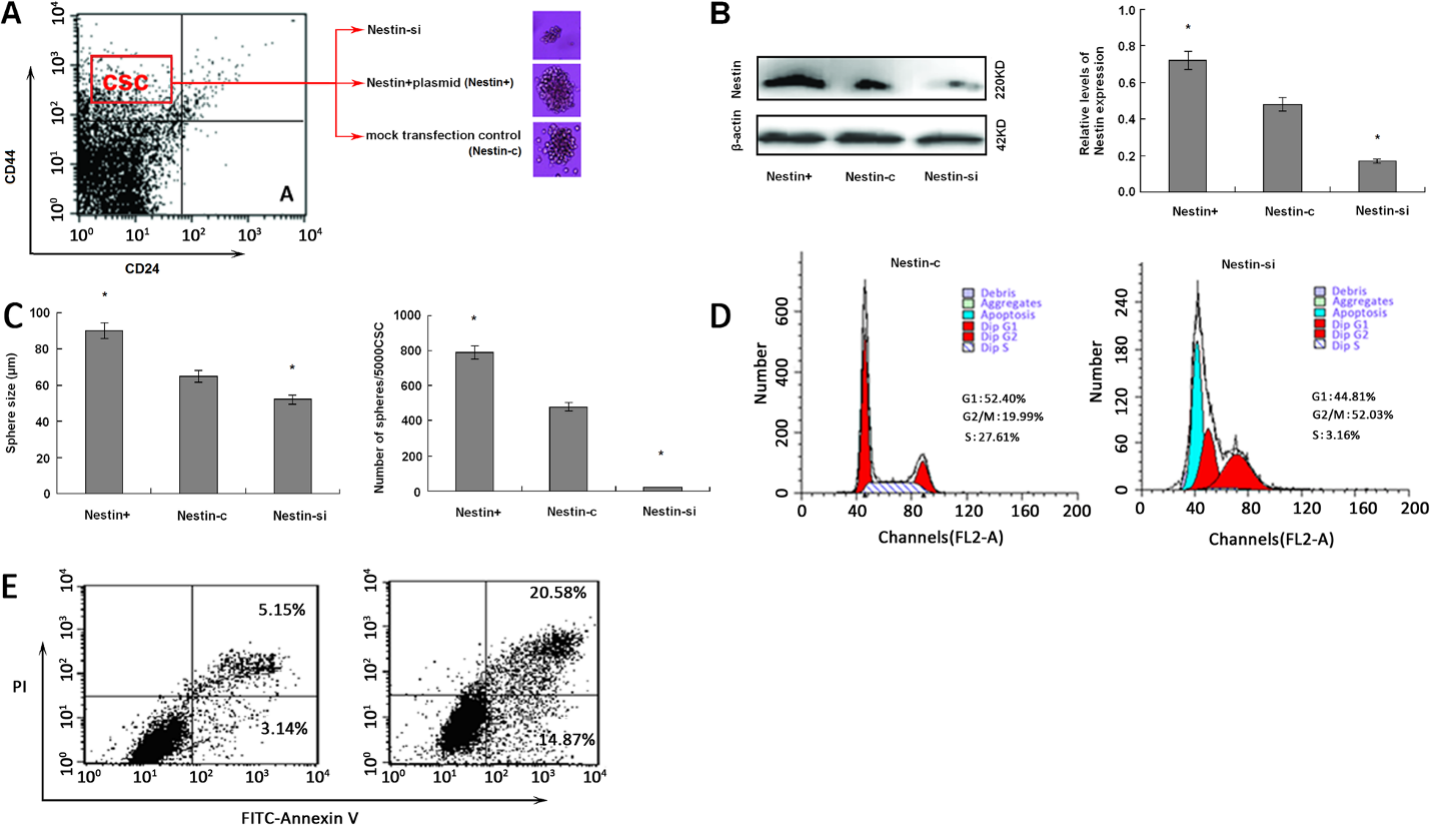
**Nestin silencing inhibits the mammosphere formation, induces cell cycle arrest at G2/M and promotes apoptosis in breast CSC.** The isolated CD44^+^CD24^−^ breast CSC from 26 specimens were transfected with control siRNA, Nestin-specific siRNA, pIRES-Nestin-EGFP, or control pIRES-EGFP to generate Nestin-c, Nestin-si, Nestin^+^ or Nestin-EGFP cells. The Nestin-c, Nestin-si, and Nestin^+^ CSC were tested for the levels of Nestin expression by western blotting and for their ability to form mammospheres *in vitro.* Their cell cycling progression and spontaneous apoptosis were determined by flow cytometry. Data are representative charts and images, and expressed as the mean ± SD of each group (n = 26 per group). **(A)**. Flow cytometric analysis of CD44^+^CD24^−^ CSC. **(B)** Western blot analysis of Nestin expression in breast CSC. Nestin-EGFP and Nestin-c control cells showed similar levels of Nestin expression (data not shown). **(C)** The mammosphere formation. **(D)** Cell cycle analysis of Nestin-si and Nestin-c breast CSC; **(E)** apoptotic Nestin-si and Nestin-c breast CSC. ^*^P <0.05 vs. the control. CSC, cancer stem cell; SD, standard deviation; siRNA, small interfering RNA.

To further examine the effect of Nestin expression on CSC, CD44^+^CD24^−^ CSC were purified and transfected with pNestin-EGFP, control siRNA, or Nestin-specific siRNA to generate Nestin^+^, Nestin-c, and Nestin-si CSC, respectively. Compared to control Nestin-c CSC, Nestin^+^ CSC expressed higher levels of Nestin while Nestin-si CSC expressed much lower levels of Nestin at 24 h posttransfection, confirming Nestin overexpression and silencing, respectively (Figure 3A and B). Nestin-si CSC did not effectively form typical mammospheres, while Nestin^+^ CSC under the same conditions formed numerous mammospheres >50 mm in diameter (Figure 3C), suggesting that increased levels of Nestin expression enhanced the proliferation of breast CSC *in vitro*. Flow cytometry analysis indicated that the percentages of Nestin-si CSC at G2/M phases and apoptotic Nestin-si CSC were significantly higher than that of Nestin-c controls (52.03% vs. 19.99% for cells at G2/M; 43.53 ± 4.78% vs. 16.24 ± 3.22% for apoptotic cells, *P* <0.01 for both, Figure 3D and E). Thus, knockdown of Nestin expression induced CSC cycle arrest at G2/M and promoted spontaneous CSC apoptosis *in vitro*, further supporting the hypothesis Nestin promoted the proliferation and survival of breast CSC.

### Nestin silencing inhibits the migration of breast CSC by downregulating the expression of EMT-related genes

**Figure 4:**
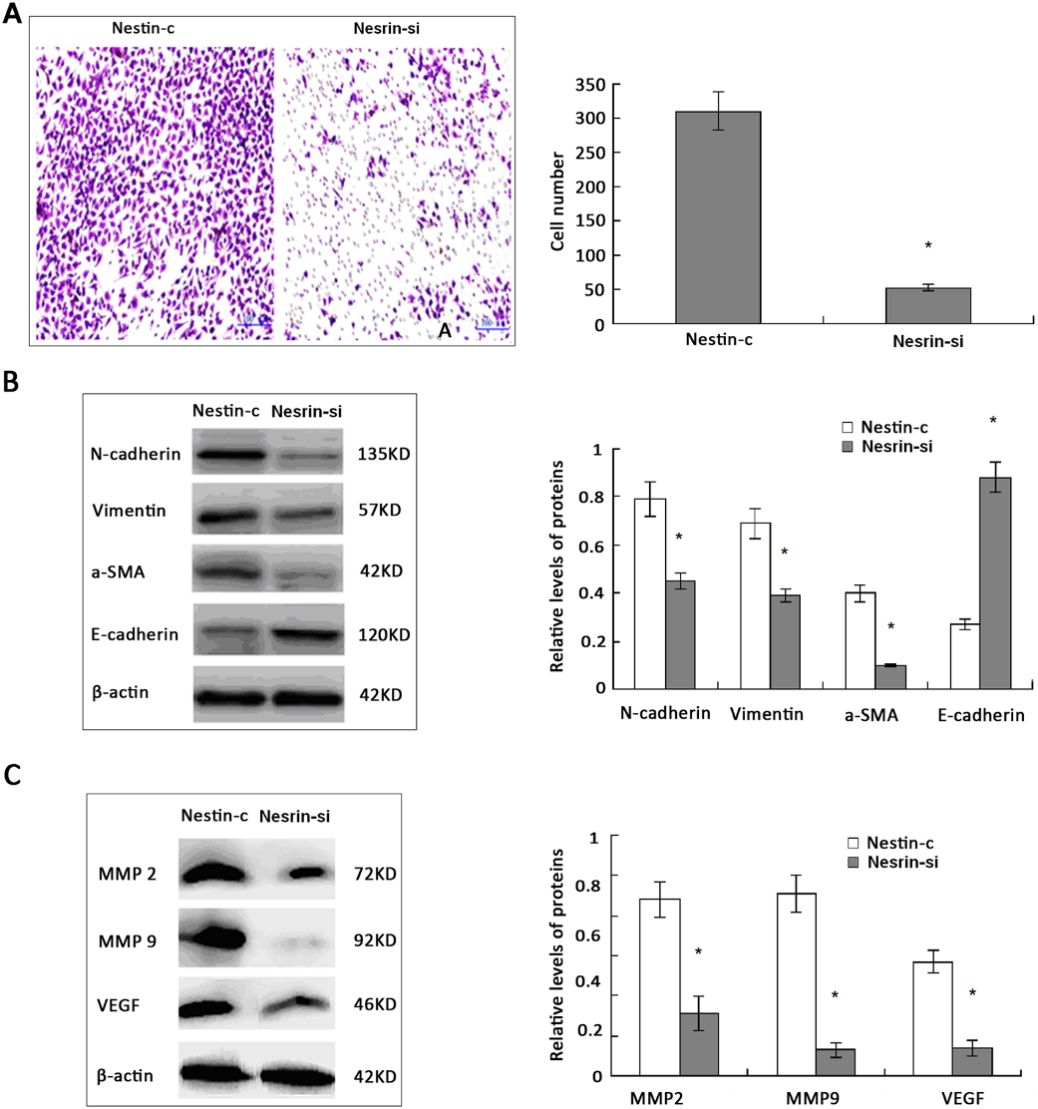
**Nestin silencing inhibits breast CSC migration by modulating the expression of EMT-related genes (A).** The migration of Nestin-si and Nestin-c breast CSC. Nestin-si and Nestin-c breast CSC were incubated in the upper chambers of transwell plates at 37 °C for 1.5 h. The migrated cells were stained with crystal violet and counted. Results are presented as mean ± SD of the numbers of migrated cells per group of cells. **(B)** and **(C)**. The relative levels of EMT-related protein expression in CSC. Nestin-si and Nestin-c breast CSC were incubated for 48 h, and the relative levels of EMT-related proteins to β-actin were determined by western blot analysis. Data shown are representative images and expressed as the mean ± SD of each target protein in each type of cells from six separate experiments. ^*^P <0.05 vs. the Nestin-c. CSC, cancer stem cell; EMT, epithelial-mesenchymal transition; SD, standard deviation.

Cancer metastasis is associated with the poor survival of patients with triple-negative breast cancer. We therefore tested the effect of Nestin silencing on the invasiveness of breast CSC using a transwell migration assay. We found that the numbers of migrated Nestin-si CSC were significantly less than control Nestin-c CSC (*P* <0.05, Figure 4A), indicating that Nestin silencing inhibited the migration of breast CSC *in vitro*.

Breast CSC migration is commonly associated with spontaneous EMT process. To investigate the potential mechanisms underlying the poor migration of Nestin-si CSC, we analyzed the relative levels of EMT-related molecules and metastatic regulators in Nestin-c and Nestin-si CSC by western blotting. The relative levels of N-cadherin, vimentin, and a-SMA expression were significantly lower in Nestin-si CSC than that in Nestin-c CSC, whereas the levels of E-cadherin were significantly higher (*P* <0.05, Figure 4B). Furthermore, significantly reduced levels of MMP2, MMP9 and VEGF were detected in Nestin-si CSC, when compared to Nestin-c CSC (*P* <0.05, Figure 4C). Together, these data indicate that knockdown of Nestin inhibited spontaneous EMT in breast CSC, contributing to their reduced invasiveness.

### Nestin silencing inhibits the Wnt/β-catenin signaling in breast CSC

**Figure 5:**
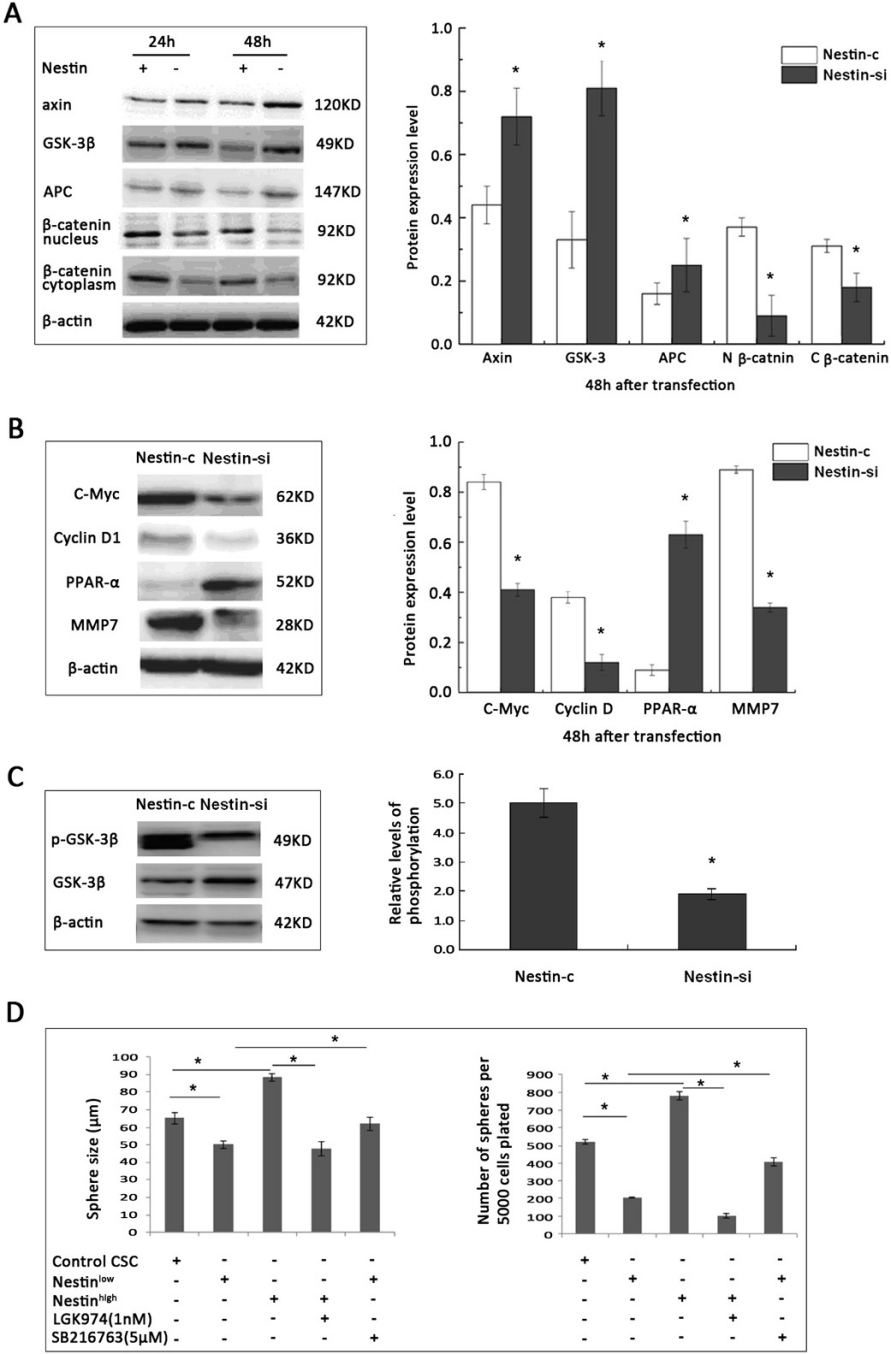
**Nestin silencing inhibits the Wnt/β-catenin signaling in breast CSC.** Nestin-si and Nestin-c breast CSC were cultured for 48 h, and the relative levels of target proteins and phosphorylation were determined by western blotting. Data are presented as the mean ± SD of the levels of target proteins vs. the β-actin control, or the phosphorylated vs. the total form, from six separate experiments. In addition, Nestin^high^ in the presence or absence of LGK974 (1 nM), Nestin^low^ in the presence or absence of SB216763 (5 μM), and control CSC were tested for their proliferation via mammosphere formation assays. Western blot analysis. **(A-C)**. Quantitative analysis of the relative levels of target proteins. **(D)** Quantitative analysis of the formed mammospheres. ^*^P <0.05, vs. the Nestin-c or control, except for specifically indicated. CSC, cancer stem cell; SD, standard deviation.

Breast CSC proliferation and migration are regulated by a range of signaling pathways, including the Wnt/β-catenin pathway. We therefore examined the impact of Nestin silencing on the Wnt/β-catenin activation in breast CSC by western blotting (Figure 5A). The relative levels of Axin, GSK-3β, and APC in Nestin-si CSC were significantly higher than that in Nestin-c CSC (*P* <0.05, Figure 5A). A similar pattern of the relative levels of mRNA transcripts of these genes was detected in Nestin-si and Nestin-c CSC (data not shown). The relative levels of nuclear and cytoplasmic β-catenin, as well as the ratio of nuclear to cytoplasmic β-catenin, were significantly lower in Nestin-si cells than in Nestin-c CSC (*P* <0.05, Figure 5A).

The relative levels of c-Myc, cyclin D1, and MMP-7 were significantly lower in Nestin-si CSC than that in Nestin-c CSC, while the relative levels of PPARa and GSK-3β expression and GSK-3β phosphorylation were significantly higher in Nestin-si CSC than that in Nestin-c CSC (*P* <0.05, Figure 5B, C). Finally, we employed the mammosphere assay to determine the importance of the Wnt/β-catenin signaling in the proliferation of CSC. We found that treatment with LGK974, an inhibitor of the PORCN-related Wnt/β-catenin signaling pathway, significantly inhibited the formation of Nestin^high^-mediated mammospheres; however, treatment with SB216763, an inhibitor of GSK-3β, significantly enhanced the formation of Nestin^low^-mediated mammospheres *in vitro* (*P* <0.05 for both, Figure 5D). These data indicate that Nestin silencing inhibits Wnt/β-catenin activation, which is crucial for the proliferation of breast CSC.

## Discussion

Previous studies have shown that Nestin expression is associated with the disease progression and poor prognosis in breast cancers, particularly for those with advanced lymph node metastasis [16],[36]. In this study, we examined Nestin expression in 150 specimens of triple-negative breast cancers, and found that 41 (27.33%) out of them were positive for anti-Nestin staining. Importantly, Nestin expression was significantly associated with poor survival in patients with triple-negative breast cancer. To the best of our knowledge, this is the first report of an association between Nestin expression and disease prognosis in Chinese patients with triple-negative breast cancer. These novel data extend previous findings in other populations, and support a role for Nestin in promoting disease progression. Therefore, Nestin may be a therapeutic target and prognostic biomarker for triple-negative breast cancer.

Nestin is known to be a neural stem cell marker, and is expressed in CSC [37], which play important roles in the progression and metastasis of breast cancer [16]. In this study, we identified that breast Nestin^high^, but not Nestin^low^ CSC had potent tumorigenicity, as evidenced by rapid mammosphere formation *in vitro* and efficient induction of solid tumors *in vivo*, consistent with our previous observations [38]. Similarly, Nestin-overexpressing Nestin^+^, but not Nestin-silenced Nestin-si CSC effectively formed mammospheres. Nestin silencing induced cell cycle arrest at G2/M phrase and promoted the spontaneous apoptosis of Nestin-si CSC. These findings extended previous observations where downregulation of Nestin expression inhibited the proliferation of melanoma cells *in vitro*[39], and the growth of implanted pancreatic tumors *in vivo*[40]. Our findings suggest that Nestin may enhance the proliferation and survival of breast CSC, contributing to the progression of triple-negative breast cancer.

Cancer metastasis is associated with poor prognosis of triple-negative breast cancer. In breast CSC, the EMT process is related to increased invasiveness and metastatic potential [26]. We observed that Nestin silencing mitigated the invasiveness of Nestin-si CSC, which was associated with significantly reduced levels of N-cadherin, vimentin and a-SMA, but increased levels of E-cadherin in breast CSC. In addition, Nestin silencing significantly reduced the relative levels of MMP2, MMP9, and VEGF in breast CSC. Together, our results indicate that Nestin silencing inhibits spontaneous EMT in breast CSC. Previous studies have showed that increased levels of EMT and expression of stemness markers are associated with progression and metastasis of invasive breast cancer, including triple-negative breast cancer [16],[36]. Our findings therefore suggest that Nestin may be a potential therapeutic target for the prevention of breast cancer metastasis. We are interested in further investigating the molecular mechanisms by which Nestin regulates the EMT process in breast CSC.

The Wnt/β-catenin pathway regulates the proliferation and tumorigenicity of stem cells.

Evidently, induction of Wnt/β-catenin overexpression enhances the tumorigenicity of breast CSC [32]. In this study, we found that knockdown of Nestin expression significantly increased the levels of Axin, GSK-3β, and APC, and the ratios of cytoplasmic to nuclear β-catenin in breast CSC. Knockdown of Nestin expression significantly reduced the relative levels of c-Myc, cyclin D1, and MMP-7, but increased the relative levels of inhibitory PPARa in breast CSC. Clearly, Nestin silencing inhibited the Wnt/β-catenin activation in breast CSC, leading to poor proliferation, invasiveness, and cell cycle arrest of CSC. Importantly, we also found that inhibition of PORCN to mitigate spontaneous Wnt secretion and the Wnt/β-catenin activation significantly reduced the proliferation of Nestin^high^ CSC, while inhibition of GSK-3 promoted the proliferation of Nestin^low^ CSC *in vitro*. These data clearly indicate that spontaneous Wnt/β-catenin activation was crucial for Nestin to promote CSC proliferation, and further supported the notion that GSK-3β is a negative regulator of the Wnt/β-catenin activation in CSC [41]. Since Nestin has no intrinsic phosphatase or protease activity, it is unlikely that Nestin directly mediates the degradation of phosphorylated GSK-3β. Indeed, many pathways, such as the ERK and PI3K/AKT pathways, can phosphorylate GSK-3β and downregulate its activity [42],[43]. It is possible that Nestin regulates the Wnt/β-catenin pathway by indirectly affecting the activity of GSK-3β in breast CSC. We are interested in further investigating how Nestin regulates GSK-3β and Wnt/β-catenin activation in breast CSC.

## Conclusions

Our data indicated that Nestin expression in breast cancer tissues was associated with poor survival of Chinese patients with triple-negative breast cancer. Furthermore, Nestin^high^, but not Nestin^low^ breast CSC, had potent tumorigenicity to form mammospheres *in vitro*, and to induce solid tumors *in vivo*. Knockdown of Nestin expression inhibited the proliferation and invasiveness, but enhanced the spontaneous apoptosis of breast CSC. Nestin silencing inhibited the spontaneous process of EMT and the Wnt/β-catenin activation in breast CSC. Our data therefore suggest that Nestin may be a potential therapeutic target for triple-negative breast cancer. Our findings may provide new insights into the roles of Nestin in regulating key cellular processes in breast CSC. Although this study examined a relatively small number of breast tumor samples, our findings extended previous observations to confirm the importance of Nestin expression in the prognosis of triple-negative breast cancer. While we did not define the precise molecular mechanisms by which Nestin promoted the Wnt/β-catenin activation in breast CSC, our data clearly indicated that spontaneous Wnt/β-catenin activation was crucial for Nestin to promote CSC proliferation, which was negatively regulated by GSK-3β. We are interested in further investigating how Nestin inhibits the growth and metastasis of breast cancers *in vivo*. Therefore, further studies are warranted to validate these findings in a larger population.

## Authors’ original submitted files for images

Below are the links to the authors’ original submitted files for images. Authors’ original file for figure 1 Authors’ original file for figure 2 Authors’ original file for figure 3 Authors’ original file for figure 4 Authors’ original file for figure 5

## Abbreviations

APC: adenomatous polyposis coli
a-SMA: a-smooth muscle actin
CSC: cancer stem cell
DAB: 3,3-diaminobenzidine
EMT: epithelial-mesenchymal transition
ER: estrogen receptor
FBS: fetal bovine serum
GSK-3β: glycogen synthase kinase-3 beta
HER2: human epidermal growth factor receptor 2
HRP: horseradish peroxidase
Ig: immunoglobulin
LIF: leukemia inhibitory factor
MMP-2: matrix metalloproteinase-2
PBS: phosphate-buffered saline
PI: propidium iodide
PPARa: peroxisome proliferator-activated receptor a
PR: progesterone receptor
qRT-PCR: quantitative reverse transcription polymerase chain reaction
SCID: severe combined immunodeficiency
siRNA: small interfering RNA
VEGF: vascular endothelial growth factor
