# Supplementary figures and images for: Nestin positively regulates the Wnt/β-catenin pathway and the proliferation, survival and invasiveness of breast cancer stem cells
Source: Breast Cancer Res. 2014 Jul 24;16:408. doi: 10.1186/s13058-014-0408-8 (PMC4220087; doi:10.1186/s13058-014-0408-8)

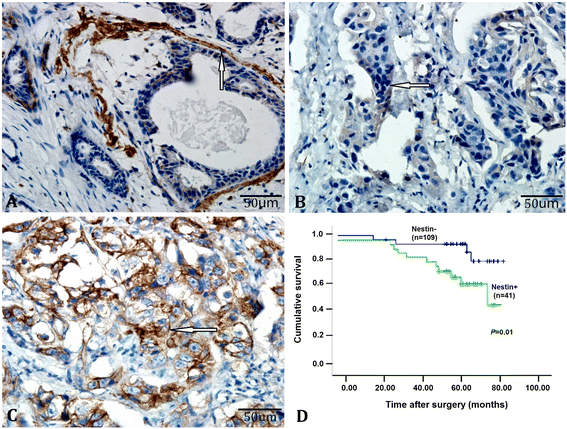

Supplement: Supplementary file 1 — Authors’ original file for figure 1 [file 13058_2014_408_MOESM1_ESM.gif]

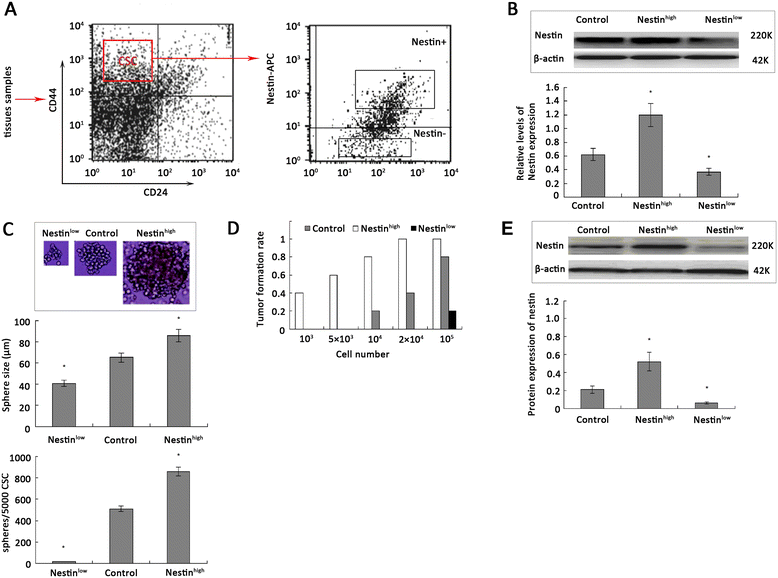

Supplement: Supplementary file 2 — Authors’ original file for figure 2 [file 13058_2014_408_MOESM2_ESM.gif]

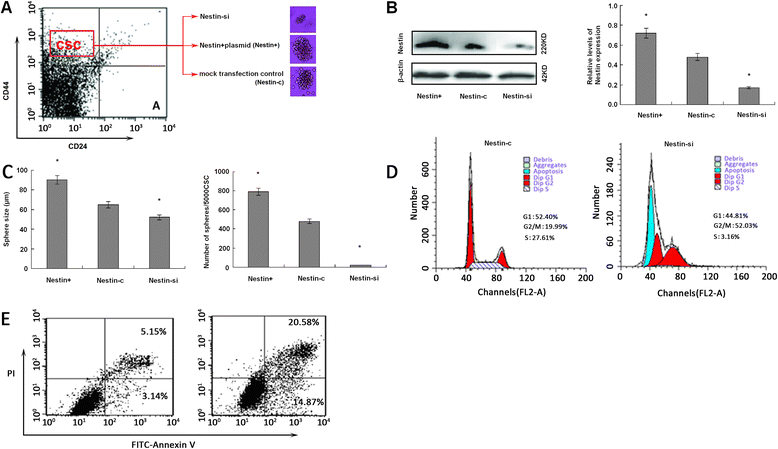

Supplement: Supplementary file 3 — Authors’ original file for figure 3 [file 13058_2014_408_MOESM3_ESM.gif]

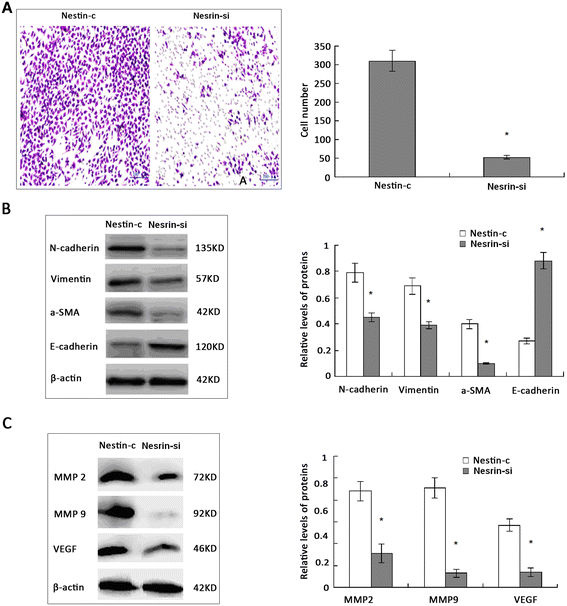

Supplement: Supplementary file 4 — Authors’ original file for figure 4 [file 13058_2014_408_MOESM4_ESM.gif]

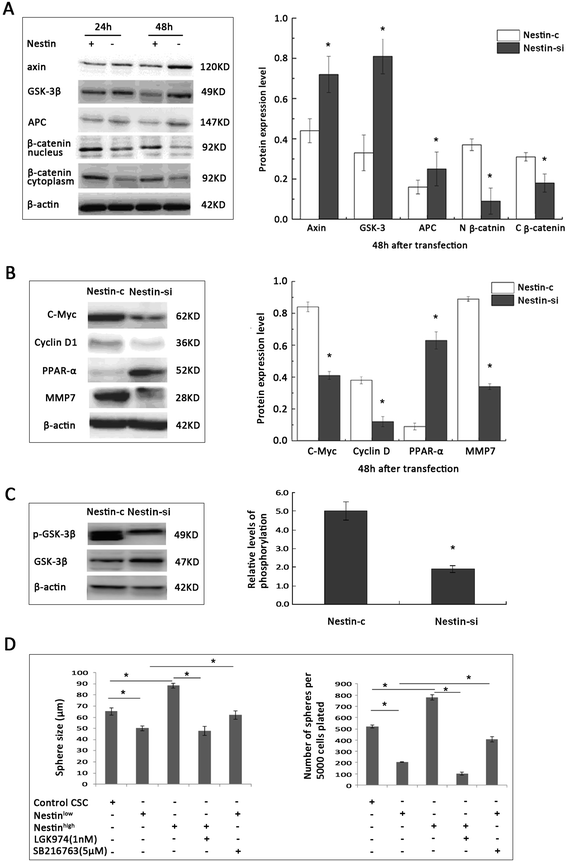

Supplement: Supplementary file 5 — Authors’ original file for figure 5 [file 13058_2014_408_MOESM5_ESM.gif]
